# Comparison of Disease Perceptions of Syphilis and AIDS Among Healthcare University Students: A Text-Mining Analysis

**DOI:** 10.7759/cureus.78533

**Published:** 2025-02-05

**Authors:** Nobuhiro Nasu, Akihiro Yokoyama, Hiromi Suzuki, Hiroaki Kataoka, Yoshiro Mori, Yuji Watanabe, Rumi Nohara, Nobuyuki Miyatake

**Affiliations:** 1 Department of Hygiene, Faculty of Medicine, Kagawa University, Miki, JPN; 2 Department of Physical Therapy, Okayama Healthcare Professional University, Okayama, JPN; 3 Department of Public Health, Graduate School of Biomedical Sciences, Tokushima University, Tokushima, JPN; 4 Department of Occupational Therapy, Faculty of Health Sciences, Okayama Healthcare Professional University, Okayama, JPN; 5 Department of Nursing, Faculty of Medicine, Kagawa University, Miki, JPN

**Keywords:** aids, qualitative analysis, syphilis, text mining, university students

## Abstract

Objective: Many studies have reported the importance of preventing sexually transmitted infections (STIs), such as syphilis and AIDS, in young adults, and it is crucial to compare and analyze impressions of syphilis and AIDS in young adults. Therefore, the present study used a text-mining analysis to compare impressions of syphilis and AIDS in students at a healthcare professional university.

Methodology: This study involved 174 students from a healthcare professional university (94 males, 80 females, aged 20.2 ± 1.2 years). Participants were surveyed on their sex, age, awareness of changes in syphilis and AIDS patient numbers in Japan over the past decade, sexual experience, and contraceptive use. Additionally, open-ended questions were asked, such as, “Please describe your impressions and thoughts about syphilis” and “Please describe your impressions and thoughts about AIDS.” The responses were then analyzed using text-mining techniques.

Results: Among all participants, 59 males (62.8%) and 47 females (58.8%) had sexual experience. Of those with sexual experience, 47 males (79.7%) and 38 females (80.8%) always used contraceptives. The text-mining analysis revealed that frequent terms for syphilis included “infection”, “image”, “nature”, “sex act”, “disease”, and “frightening”, while those for AIDS were “infection”, “image”, “disease”, “impression”, “be cured”, and “frightening”. The term “be cured” was a characteristic word for AIDS. The term “frightening” was common for syphilis in females and for AIDS in males and was also notable for syphilis and AIDS in those without sexual experience or those who did not always use contraceptives.

Conclusions: The comparison of impressions of syphilis and AIDS in students at a healthcare professional university using a text-mining analysis revealed differences between the two diseases. The term “be cured” was a characteristic word for AIDS, while “frightening” was common for syphilis in females and for AIDS in males.

## Introduction

Syphilis is a major sexually transmitted infection (STI) caused by Treponema pallidum [1]. Although treatable, the lack of awareness of, screening for, and education on syphilis has resulted in a high mortality rate worldwide [1]. According to the World Health Organization (WHO), the number of infected adults with syphilis globally in 2022 was 8 million, and it has become a public health challenge [2]. In Japan, syphilis has been increasing since 2012, with a sharp increase after 2021, and approximately 13,000 cases, a record number, were reported in 2022 [1,3]. It commonly affects males in their 20s to 40s and females in their 20s in Japan [3].

AIDS is an immune system disease caused by HIV infection [4]. HIV remains a major public health issue, and transmission is ongoing worldwide [4]. According to the Joint United Nations Program on HIV/AIDS, an estimated 39.9 million individuals were reported to be infected worldwide in 2023 [5]. The number of new infections is 1.3 million, which is a 38% decrease from 2.1 million in 2010 [5]. In Japan, the annual number of new HIV cases reported in 2022 was 632 (903 in 2019, 750 in 2020, and 742 in 2021) and the annual number of new AIDS patients in 2022 was 252 (333 in 2019, 345 in 2020, and 315 in 2021), with a decrease since 2016 [6]. The number of newly reported HIV-infected persons per year is slightly higher among those in their 20s and 30s, while the number of newly reported AIDS patients per year is slightly higher among those in their 30s and 40s [6]. Therefore, syphilis and AIDS are more common among young adults. Previous studies emphasized the importance of preventing STIs, such as syphilis and AIDS, among young adults [7-10]. Therefore, disseminating accurate information about STIs and preventive behaviors is crucial for preventing the future spread of these diseases.

Quantitative studies assessed knowledge and impressions of STIs, such as syphilis and AIDS, mainly using questionnaires [11-15]. Diseases have also been examined using a qualitative analysis, such as text mining. Okuhara et al. analyzed newspaper articles to investigate the cause of decreases in vaccination before and after the human papillomavirus (HPV) vaccination crisis in Japan using a text-mining analysis [16]. Furthermore, Falissard et al. examined the effects of atopic dermatitis on daily activities and found that both physical and emotional factors played an important role [17]. Mori et al. compared impressions of COVID-19 vaccination and influenza vaccination in Japan using a text-mining analysis of social media (Twitter®) and reported marked differences [18]. Yokoyama et al. also compared impressions of COVID-19 vaccinations stratified by the number of vaccinations among healthcare professional university students in Japan. Accurate information on safety for unvaccinated students and side effects for vaccinated students was considered to be important among students [19]. Based on these findings, qualitative analyses, including a text-mining analysis, provide useful information that differs from that of quantitative analyses. In addition, the difference in impressions between syphilis and AIDS might influence preventive behaviors or stigma differently, and clarifying this difference would provide a useful strategy for preventing these diseases. However, impressions of syphilis and AIDS have not yet been compared in young adults in Japan using a text-mining analysis.

Therefore, to prevent STI in Japanese young adults in the future, we herein compared and analyzed impressions of syphilis and AIDS in healthcare professional university students in Okayama, Japan, for the first time.

## Materials and methods

Subjects

**Figure 1:**
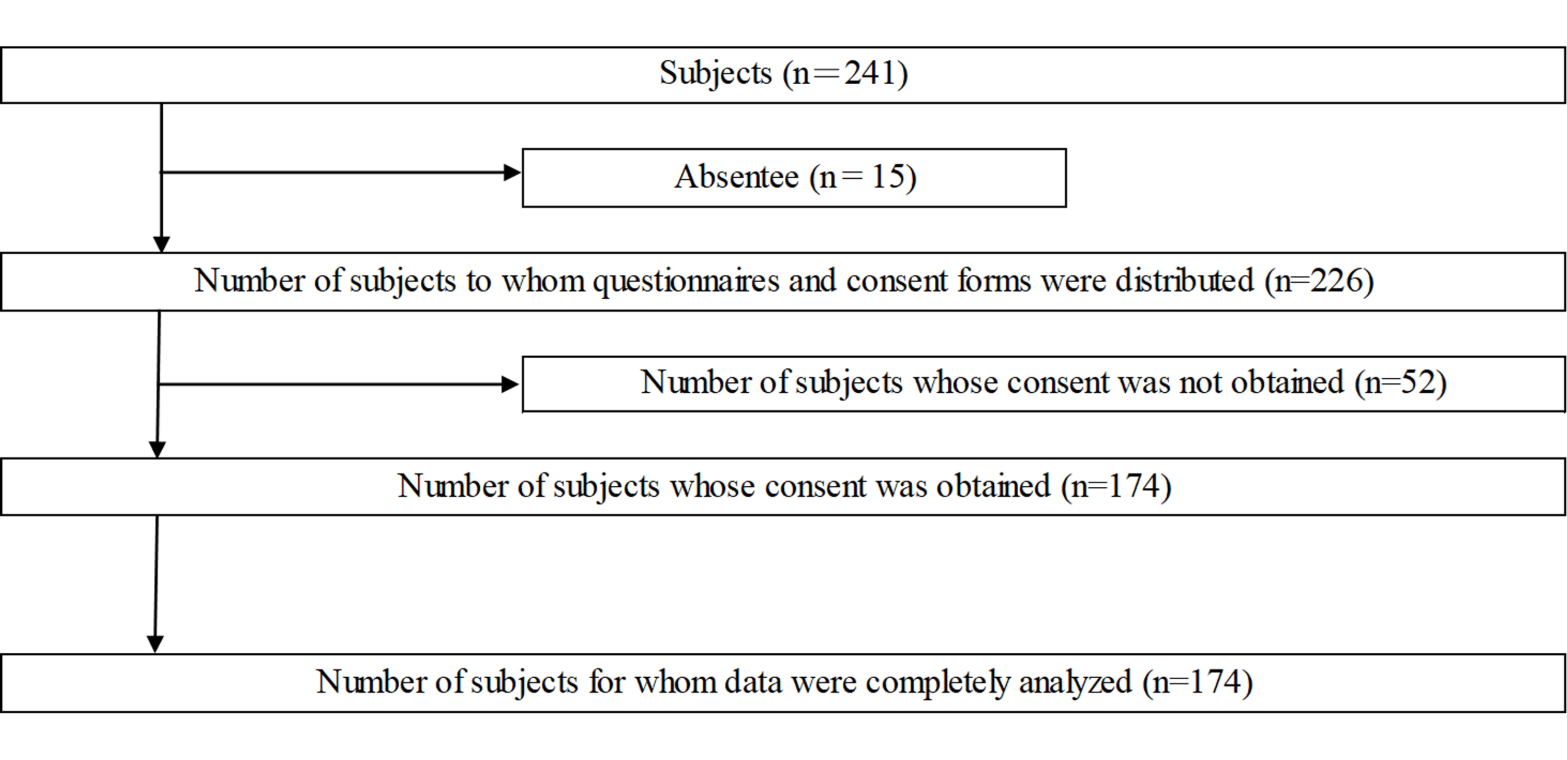
Selection process for the target population. Image credit: Nobuhiro Nasu.

A total of 174 students (94 males, 80 females, aged 20.2 ± 1.2 years) out of 241 students who met the following criteria were enrolled in this cross-sectional study: (1) healthcare professional university students in Okayama Prefecture, Japan, and (2) those who provided written informed consent for the survey (Figure 1).

Questionnaire

A self-reported questionnaire, including questions such as “Please describe your impressions and thoughts about syphilis” and “Please describe your impressions and thoughts about AIDS,” was completed between June 17, 2024, and July 29, 2024. In addition to information on sex and age, responses to the following questions were obtained: “Which of the following applies to the number of syphilis cases in Japan in the last 10 years?” (increasing/decreasing), “Which of the following applies to the number of AIDS patients in Japan in the last 10 years?” (increasing/decreasing), “Have you ever had sexual intercourse?” (yes/no), and “For those who answered ‘yes,’ do you use contraceptives during sexual intercourse to prevent infection?” (always/occasionally/do not use). These surveys were administered using QR codes and Google Forms® [20]. In addition, analysts did not have access to respondents’ e-mail addresses or other personal information. Questions were answered from students' smartphones. The questionnaire took 5 to 10 minutes to complete.

Statistical analysis

Data on sex and age were expressed as the number of subjects and percentages. Questions about syphilis and AIDS were presented as numbers and percentages for both males and females and also separately. Perceptions of increases or decreases in syphilis and AIDS patients in Japan in the past 10 years, as well as the presence or absence of sexual experience, were analyzed using a chi-square test. Fisher’s exact probability test was used to analyze the presence or absence of contraceptive use, with *P* < 0.05 being significant. These analyses were performed using JMP Pro 17 (SAS Institute Inc., Cary, NC).

The self-reported questionnaire on impressions of syphilis and AIDS was analyzed using text-mining software (KH Coder 3.0, Koichi Higuchi, Tokyo, Japan), as previously described [21,22]. Words were automatically extracted from the text collected via Google Forms®, and a list of frequently used words was created using KH Coder. KH Coder was run with default settings, including the morphological analysis dictionary, to minimize bias in the analyst's opinions. A correspondence analysis was then conducted to visualize the relationships between words in scatter plots, analyzed by sex (syphilis/AIDS), the presence or absence of sexual experience (syphilis/AIDS), and the presence or absence of contraceptive use (syphilis/AIDS). Extracted words were translated into English using DeepL® translation (Cologne, Germany) [23]. DeepL® translation and ChatGPT were used for English text proofreading.

## Results

Clinical profiles are summarized in Table 1. The enrolled students comprised 94 males (54.0%) and 80 females (46.0%), with a mean age of 20.2 ± 1.2 years.

**Table 1:** Clinical profiles of enrolled subjects (n = 174).

Clinical profile	Number of subjects, *n* (%)
Sex	
Male	94 (54)
Female	80 (46)
Age (years)	
18	11 (6.3)
19	37 (21.3)
20	53 (30.5)
21	51 (29.3)
22	20 (11.5)
23	1 (0.6)
27	1 (0.6)
Average age (years)	20.2 ± 1.2

 Table 2 shows responses to questions on syphilis and AIDS. Regarding responses to “Which of the following applies to the number of syphilis cases in Japan in the last 10 years?” and “Which of the following applies to the number of AIDS patients in Japan in the last 10 years?”, the number of subjects who answered “yes” to syphilis and AIDS was higher than those who answered “no”, with no sex difference. Among all participants, 59 males (62.8%) and 47 females (58.8%) had sexual experience. Of those with sexual experience, 47 males (79.7%) and 38 females (80.8%) always used contraceptives. No significant sex differences were observed in either case.

**Table 2:** Responses to questions about syphilis and AIDS. *Chi-square test. **Fisher's exact probability test.

Question	Answer	Total	%	Male	%	Female	%	P	Chi-square value
Which of the following applies to the number of syphilis cases in Japan in the last 10 years? (*n* = 174)	Increasing	145	83.3	79	84.0	66	82.5	0.786*	0.074
	Decreasing	29	16.7	15	16.0	14	17.5		
Which of the following applies to the number of AIDS patients in Japan in the last 10 years? (*n* = 174)	Increasing	142	81.6	73	77.7	69	86.2	0.144*	2.125
	Decreasing	32	18.4	21	22.3	11	13.8		
Have you ever had sexual intercourse before? (*n* = 174)	Yes	106	60.9	59	62.8	47	58.8	0.588*	0.293
	No	68	39.1	35	37.2	33	41.2		
Those who answered “yes”, do you use contraceptives during sexual intercourse to prevent infection? (*n* = 106)	Always	85	80.2	47	79.7	38	80.8	0.370**	
	Occasional	17	16.0	11	18.6	6	12.8		
	Do not use	4	3.8	1	1.7	3	6.4		

We then performed a text-mining analysis (Tables 3-4). Regarding syphilis, 2,099 words were extracted, and the most frequent word was “infection” (66 times), followed by “image” (30 times), “nature” (29 times), “sex act” (28 times), “disease” (22 times), and “frightening” (19 times) (Table 3). Concerning AIDS, 2056 words were extracted and the most frequent word was “infection” (78 times), followed by “image” (36 times), “disease” (27 times), “impression” (20 times), “be cured” (19 times), and “frightening” (18 times) (Table 4). The term “be cured” was characteristic of AIDS.

**Table 3:** Frequently used words for syphilis (2,099 total words).

Rank	Extracted word	Number of times	%
1	Infection	66	3.1
2	Image	30	1.4
3	Nature	29	1.4
4	Sex act	28	1.3
5	Disease	22	1.0
6	Frightening	19	0.9
7	Person	18	0.9
8	Impression	17	0.8
9	Hear	16	0.8
10	Be understood	15	0.7

**Table 4:** Frequently used words for AIDS (2,056 total words).

Rank	Extracted word	Number of times, *n*	%
1	Infection	78	3.8
2	Image	36	1.8
3	Disease	27	1.3
4	Impression	20	1.0
5	Be cured	19	0.9
6	Frightening	18	0.9
7	Think	17	0.8
8	Sex act	17	0.8
9	Immunity	17	0.8
10	Person	15	0.7

**Figure 2:**
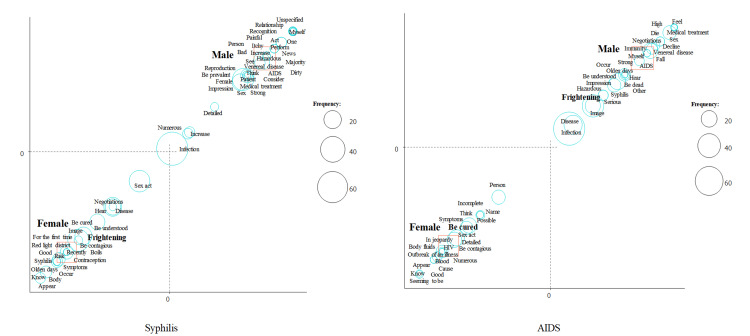
Correspondence analysis of syphilis and AIDS stratified by sex.

**Figure 3:**
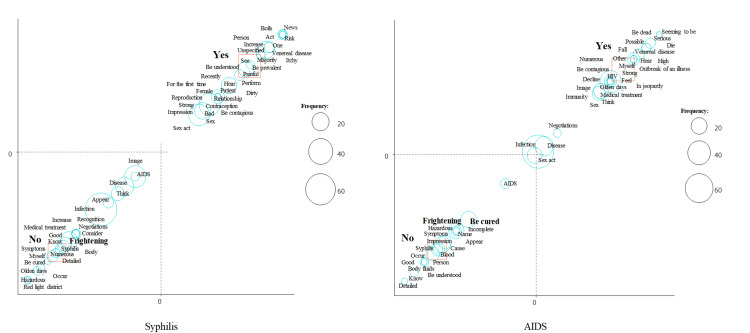
Correspondence analysis of syphilis and AIDS stratified by sexual intercourse experience.

**Figure 4:**
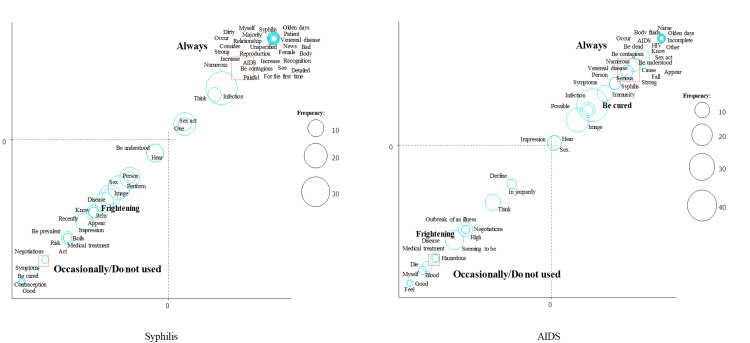
Correspondence analysis of syphilis and AIDS stratified by contraceptive use.

We then performed a correspondence analysis (Figures 2-4). We focused on the word “frightening.” “Frightening” was a characteristic word for syphilis in females and for AIDS in males (Figure 2), for syphilis and AIDS in participants without sexual experience (Figure 3), and for syphilis and AIDS in participants who do not always use contraceptives (Figure 4).

## Discussion

By using a text-mining analysis, we herein examined differences in impressions of syphilis and AIDS in healthcare professional university students in Okayama Prefecture, Japan. The word “frightening” was a characteristic word for AIDS among males and syphilis among females. It was also commonly associated with both syphilis and AIDS among participants without sexual experience and those who do not consistently use contraceptives.

Regarding their recognition of the number of patients with syphilis in the last 10 years, more than 80% of students were aware that this number is increasing (males, 84.0%; females, 82.5%). Males (77.7%) and females (86.2%) both recognized that the number of patients with AIDS is increasing. In Japan, the number of syphilis patients is increasing, while that of AIDS patients is decreasing [1,3,6]. This difference may be due to the rapid increase in the number of syphilis patients in recent years being widely reported by the media, which has raised awareness of this infectious disease crisis. However, there has been a decrease in information on AIDS and students cannot easily access the latest information. To resolve this discrepancy in impressions, it is important to provide the latest data and accurate information.

The WHO Regional Office for Europe reported that the percentage of young individuals (15 years old) using contraceptives during sexual activity decreased from 70% to 61% in males and from 63% to 57% in females between 2014 and 2022 [24]. In a survey of Portuguese university students, Santos et al. revealed that 39.4% of students always used contraceptives [25]. In the present study, approximately 80% of students of both sexes who had sexual experience said that they always used contraceptives, which was higher than the contraceptive use rate reported in previous studies [24,25]. Although the risk of STI increases without the use of contraceptives, approximately 20% of students do not always use contraceptives. Crosby et al. showed that the risk of STI may be significantly reduced by the correct use of contraceptives [26]. Therefore, it is necessary to provide sex education and appropriate information on contraceptive use to the 20% of students who do not always use contraceptives.

The text-mining analysis revealed that the phrase “be cured” was a characteristic word for AIDS. The terms “be cured” included “cannot be cured easily” and “cannot be cured” and also included “be cured by medications.” In the study by Subbarao et al., 30.5% of university students stated that there is currently no cure for HIV/AIDS, 31.4% stated that HIV/AIDS may be cured, and 30.8% stated that they do not know if there is a cure for HIV [14]. Although accurate knowledge of AIDS may be insufficient, recent advances in medications have spread awareness that AIDS may be managed similarly to many other diseases and also that it is now more easily recognized as curable. In addition, since awareness of AIDS is higher than that of other STIs, students’ awareness of AIDS may be shifting from prevention to treatment.

The stratification of students according to sex showed that “frightening” was fundamentally characteristic of syphilis in females and AIDS in males. This result suggests that impressions may differ by sex. Carvalho and Araújo reported that males had less knowledge of syphilis than females [12], and Morales et al. showed that females negotiated contraceptive use and avoided risky sexual behavior more than males because of their more detailed knowledge of syphilis than males [27]. Therefore, more “frightening” impressions are likely to be held by females who understand the serious health risks and social prejudices of syphilis. Another possible reason why the “frightening” impression of syphilis was stronger in females was related to concerns about its future impact on reproduction and mother-to-child transmission. In a study on pregnant females, subjects had more detailed knowledge of the mother-to-child transmission of syphilis rather than other infectious diseases [28]. Females may be more sensitive to health issues related to sex and pregnancy, which may affect their impression of the disease as “frightening.” In the present study, the term “frightening” was a characteristic word for AIDS in males. A study by Nishi, which surveyed images and attitudes toward AIDS in university students, reported negative images of hopelessness and fear. Regarding attitudes, males had more “aversive” and “prejudicial” attitudes than females [29]. Therefore, the sex-specific prevention of STI may be needed.

The stratification of students based on sexual experience revealed that the term “frightening” was predominantly characteristic of both syphilis and AIDS among students without sexual experience. In previous studies, the main sources of information on STIs were the Internet and school classes (teachers) [10,13-15]. In addition, confusion and assumptions were attributed to the lack of proper education on STI [30]. This education emphasizes the risk and seriousness of infection, which amplifies anxiety and fear. Therefore, in education to prevent STIs, the provision of specific and practical information needs to be prioritized over fear-mongering.

The stratification of students by contraceptive use showed that the term “frightening” was a characteristic word for both syphilis and AIDS in those who did not always use contraceptives. This result indicates that fear of STIs is not always associated with preventive behavior. Paloga et al. showed that fear and knowledge of STIs (HIV and syphilis) were not linked to preventive perceptions of STIs [11]. Although students had some knowledge of STI, their preventive behaviors were inadequate [15]. Rather than education that appeals to fear, an approach that encourages practical and sustainable preventive behaviors is needed. Specifically, we need to emphasize the benefits of contraceptive use and explain how to use them.

The present results suggest that appropriate sex education, including the proper use of contraceptives, is important for students who have never had sex and also for students who have had sex without using contraceptives. Previous studies demonstrated that proper sex education in schools and medical facilities increased the contraceptive use rate and improved knowledge and attitudes toward STI [7-10].

Several limitations need to be addressed. This was a cross-sectional study at one healthcare professional university in Okayama Prefecture, Japan. Furthermore, we only analyzed 174 of 241 subjects. Moreover, healthcare professional university students enrolled in this study were considered to be more health-conscious than average young adults in Japan. Another limitation is that some of the questions, including items related to sex, may not have been answered accurately. Nevertheless, the results obtained herein provide useful information for education on and the prevention of STIs, including syphilis and AIDS, in the future.

## Conclusions

In the present study, we analyzed impressions of syphilis and AIDS in healthcare professional university students in Okayama Prefecture, Japan. The term “frightening” was a characteristic word for AIDS in male students and for syphilis in female students. It was also commonly used for both syphilis and AIDS in students with no sexual experience and those who do not always use contraceptives. This result indicates that their impressions are connected to their backgrounds and experiences, that is, better sex education could help correct misunderstandings and reduce fear of these diseases. In addition, appropriate sex education, including the proper use of contraceptives, is particularly important for students who have never had sex and also for students who have had sex without using contraceptives.
